# Vayu bubble continuous positive airway pressure is a promising solution with favorable treatment outcomes for respiratory distress syndrome in newborns: a qualitative study in Bangladesh

**DOI:** 10.3389/fped.2024.1359406

**Published:** 2024-04-29

**Authors:** Goutom Banik, M. A. Halim, Abu Sayeed Md. Abdullah, Irtifa Oishee, Carolyn Boyce, Sanjoy Kumer Dey, Md Abdul Mannan, Sadeka Choudhury Moni, Mohammad Kamrul Hassan Shabuj, Ismat Jahan, Rumpa Mani Chowdhury, Sharmin Afroze, Steve Wall, Mohammod Shahidullah

**Affiliations:** ^1^ Health and Nutrition Sector, Save the Children Bangladesh, Dhaka, Bangladesh; ^2^ Centre for Injury Prevention and Research, Dhaka, Bangladesh; ^3^ Save the Children Federation, Inc., Fairfield, CT, United States; ^4^ Department of Neonatology, Bangabandhu Sheikh Mujib Medical University, Dhaka, Bangladesh; ^5^ Department of Neonatology, Dr. M R Khan Shishu Hospital & Institute of Child Health, Dhaka, Bangladesh

**Keywords:** newborns, respiratory distress syndrome, bubble CPAP, prematurity, low birth weight, neonatal mortality, Bangladesh

## Abstract

**Background:**

According to Bangladesh Demographic and Health Survey (2022), neonatal mortality, comprising 67% of under-5 deaths in Bangladesh, is significantly attributed to prematurity and low birth weight (LBW), accounting for 32% of neonatal deaths. Respiratory distress syndrome (RDS) is a prevalent concern among preterm and LBW infants, leading to substantial mortality. The World Health Organization (WHO) recommends bubble continuous positive airway pressure (bCPAP) therapy, but the affordability and accessibility of conventional bCPAP devices for a large number of patients become major hurdles in Bangladesh due to high costs and resource intensiveness. The Vayu bCPAP, a simple and portable alternative, offers a constant flow of oxygen-enriched, filtered, humidified, and pressurized air. Our study, conducted in five health facilities, explores the useability, acceptability, and perceived treatment outcome of Vayu bCPAP in the local context of Bangladesh.

**Methods:**

A qualitative approach was employed in special care newborn units (SCANUs) of selected facilities from January to March 2023. Purposive sampling identified nine key informants, 40 in-depth interviews with service providers, and 10 focus group discussions. Data collection and analysis utilized a thematic framework approach led by trained anthropologists and medical officers.

**Results:**

Service providers acknowledged Vayu bCPAP as a lightweight, easily movable, and cost-effective device requiring minimal training. Despite challenges such as consumable shortages and maintenance issues, providers perceived the device as user-friendly, operable with oxygen cylinders, and beneficial during referral transportation. Treatment outcomes indicated effective RDS management, reduced hospital stays, and decreased referrals. Though challenges existed, healthcare providers and facility managers expressed enthusiasm for Vayu bCPAP due to its potential to simplify advanced neonatal care delivery.

**Conclusions:**

The Vayu bCPAP device demonstrated useability, acceptability, and favorable treatment outcomes in the care of neonates with RDS. However, sustained quality service necessitates continuous monitoring, mentoring and retention of knowledge and skills. Despite challenges, the enthusiasm among healthcare providers underscores the potential of Vayu bCPAP to save lives and simplify neonatal care delivery. Development of Standard Operating procedure on Vayu bCPAP is required for systematic implementation. Further research is needed to determine how the utilization of Vayu bCPAP devices enhances accessibility to efficient bCPAP therapy for neonates experiencing RDS.

## 1 Introduction

Globally, almost one million newborns die from complications of prematurity every year. The most common severe preterm complication is respiratory distress syndrome (RDS), a primary cause of mortality in premature newborns. Worldwide, approximately 11% of all live births occur before 37 weeks of gestation (1), with preterm birth rates increasing. Survival is improving, especially in well-resourced settings, primarily due to improved healthcare and widely available and effective technology (2).

In Bangladesh, approximately 604,000 babies are born prematurely each year (before 37 completed weeks gestation), with an annual preterm birth rate of 14% (3). Complications of preterm birth account for 23,600 direct preterm child deaths per year, many from respiratory conditions (4). The bubble continuous positive airway pressure (bCPAP) device is included in Bangladesh's National Newborn Health Program (NNHP) as a key intervention of the special care newborn unit (SCANU) program to provide quality facility-based care to newborns with complications (5).

Newborns and young infants with respiratory distress from other conditions, such as Meconium Aspiration Syndrome and pneumonia, also are at heightened risk of death (6). To improve the outcomes of newborns and infants with respiratory distress, continuous positive airway pressure (CPAP) is extremely effective in well-resourced settings with reported 65% mortality decrease in RDS (7). Widely used in high-income settings, CPAP use in less-resourced settings—where needed most—is limited due to the high cost, reliance on compressed air and continuous electricity, maintenance, availability of consumables, and lack of necessary skills (8). The United States Food and Drug Administration (FDA) has granted Emergency Use Authorization (EUA) for a life-saving medical device to provide respiratory support to patients with respiratory distress in these less-resourced settings. The device, called the Vayu bubble CPAP (Vayu bCPAP), developed by Vayu Global Health, is an effective means to help save newborns and infants from preventable respiratory distress deaths. It is high-quality, easy to use, and extremely affordable, with production costs lower than other comparable devices (9, 10). The device provides adjustable inspired concentrations of oxygen (FiO2), flow rates, and pressures, as well as humidification comparable to a gold standard bCPAP device, yet does not require compressed air or electricity (11).

To manage RDS, respiratory support is needed, but necessary oxygen concentration, flow rates, and pressure are often not available in low-resource settings worldwide. In Bangladesh, commercial bCPAP devices remain out of reach for many newborns with respiratory distress due to costs, challenges for healthcare providers to achieve and maintain the requisite skills, and difficulties in maintaining well-functioning bCPAP equipment. Other common barriers include intermittent losses of electricity and lack of compressed air required for commercial bCPAP devices (10).

Considering the high maintenance issues of the other available bCPAP devices, we aimed to conduct a study to determine whether the use of the Vayu bCPAP and oxygen blender is useable and acceptable by health care providers (HCPs) in SCANUs as part of routine care for newborns with respiratory distress, primarily for preterm newborns in the local context of Bangladesh. Before initiating the study, a total of 28 Vayu bCPAP and oxygen blender devices were distributed and installed in study facilities. We organized a national-level workshop at Bangabandhu Sheikh Mujib Medical University (BSMMU) in August 2022 to sensitize the national- and local-level stakeholders and develop a plan for appropriate implementation of Vayu bCPAP in the study facilities. We also established a group of national-level master trainers for periodic monitoring and mentoring support for service providers in the study facilities. Following that, we trained a total of 50 service providers from the target facilities in using Vayu bCPAP. Periodic monitoring visits from the national level as well as mentoring were ongoing to encourage discussion and collaboration in addressing implementation challenges at the facility level. Over the implementation period (September 2022-March 2023), a total of 410 newborns received treatment with Vayu bCPAP in the SCANU settings of the study facilities.

## 2 Methods

### 2.1 Study design and setting

The study design involved qualitative methods to gather and analyze data. Qualitative research focuses on exploring the usability, acceptability and perceived treatment outcome in depth using different qualitative approaches of data gathering. We selected five different types of health facilities in consultation with Bangladesh's NNHP of the Directorate General of Health Services (DGHS) to implement the research. The five facilities included a medical university—BSMMU in Dhaka; a medical college hospital—Sylhet Medical College Hospital (SOMCH) in Sylhet; a district hospital—Lakshmipur District Hospital (LDH) in Lakshmipur; a specialized hospital—Mohammadpur Fertility Services and Training Center (MFSTC) in Dhaka; and a private specialized hospital—Dr. MR Khan Shishu Hospital (MR Khan) in Dhaka. Each selected facility represented a unique setting within Bangladesh's healthcare landscape, ranging from tertiary care institutions like medical universities to specialized and private hospitals. By including diverse types of facilities, the study aimed to capture a broad spectrum of experiences, perspectives, and practices related to the implementation of Vayu bCPAP in Bangladesh.

### 2.2 Sampling and sample

The sampling process employed in this study utilized purposive sampling. Purposive sampling involves selecting individuals or groups who possess specific characteristics or experiences relevant to the research objectives. In this case, the researchers purposefully selected participants who could provide valuable insights on the usability, acceptability, and perceived treatment outcome of Vayu bCPAP. Data collection was ongoing before achieving data saturation. Data saturation refers to the point at which no new information or insights emerge from additional data collection, indicating that a comprehensive understanding of the topic has been attained. We conducted nine key informant interviews (KIIs) with facility managers, 40 in-depth interviews (IDIs) with HCPs, and 10 focus group discussions (FGDs) with nurses and support staff.

### 2.3 Data collection and quality assurance

The primary investigator (PI) and Co-PI of the study drafted, reviewed, and finalized the interview guidelines and consent forms. The guidelines were developed based on the following issues: relevance, efficiency, effectiveness, impact, sustainability, and coverage, capacity to use the following training, ease of use (assembly, application, monitoring, maintenance, and troubleshooting), ability to integrate use into care processes, facilitators and barriers to use (including suggested improvements), positive or negative effects on treatment outcomes, positive and negative attributes of the Vayu bCPAP system, and lessons learned that can be replicated in other facilities.

We recruited a total of four data collectors, including two anthropologists (one male and one female) and two medical doctors (one male and one female). These data collectors underwent a comprehensive two-day training on data collection, which included mock tests and role-playing activities. The training was designed to prepare them for qualitative data collection. Two teams carried out the data collection process. Each team consisted of one anthropologist and one doctor, one male and one female. Together, they worked to collect data and ensure the success of the project.

Data were collected through FGDs, IDIs, and KIIs. The teams conducted a total of 40 IDIs, five KIIs, and 10 FGDs within the five study facilities, and four KIIs at the national level (Table 1). All interviews and focus group discussions were performed in a private area, using audio recordings and paper forms by the research team.

**Table 1:** Respondents for the study's KIIs, FGDs, and IDIs in each facility.

Facility	KIIs (9)	FGDs (10)	IDIs (40)
MR Khan	Assistant Professor	FGD-1: Ward Boy-6 FGD-2: Senior Staff Nurse -6	Senior Staff Nurse -2/Technician-1 Medical Officer-3/Consultant-1/Ward Boy 1
MFSTC	Head of the Department	FGD-1: Senior Staff Nurse -6 FGD-2: Senior Staff Nurse -6	Technician-1/Senior Staff Nurse -1 Midwife-2/Medical Officer-2/Consultant-2
BSMMU	Chairman of Neonatology Department	FGD-1: Support Staff-6 FGD-2: Senior Staff Nurse -6	Senior Staff Nurse-3/Technician-1 Senior Medical Officer-2/Resident Medical Officer-2
SOMCH	Pediatric Consultant (In-charge)	FGD-1: Senior Staff Nurse -6 FGD-1: Senior Staff Nurse -6	Assistant Register-1 Indoor Medical Officer-1/Senior Staff Nurse-6
LDH	Pediatric Consultant (In-charge)	FGD-1: Senior Staff Nurse (Pediatric)-5 FGD-1: Senior Staff Nurse (SCANU)-5	Consultant-1 Medical Officer-1/Senior Staff Nurse-6
National	Program Manager, Integrated Management of Childhood Illness (IMCI)-1 Deputy Program Manager, IMCI-3		

A quality control team consisting of senior research team members was deployed for quality control checking of the study data. Quality control checking was designed to physically verify whether the data collectors effectively completed the interviews and FGDs (i.e., interviewed the right respondents and asked the right questions). The quality control team undertook quality control checking both in the presence and absence of the interviewing team. After data collection, the PI and Co-PI supervised processing of all the qualitative findings.

### 2.4 Data analysis

After conducting interviews and focus group discussions, the audio recordings of these sessions were transcribed into written text. Two anthropologists were responsible for independently transcribing the recordings. This approach helps ensure accuracy and completeness in capturing the content of the discussions. Transcription was made in Bangla and subsequently translated into English. Using thematic analysis (12), major themes were identified and coded. Two research assistants coded the transcriptions independently. Coding involves systematically labeling and categorizing segments of the text based on recurring themes. The process examines the content of the transcriptions to identify ideas, perspectives, or experiences pointed repeatedly related to the use of the Vayu bCPAP device in managing respiratory distress syndrome in neonates. This step allows researchers to organize the data and identify key topics for analysis. After sorting and categorizing the responses, we chose excerpts from the transcripts to illustrate the summary statements, which were used to validate the findings. This rigorous process ensured systematic analysis and interpretation of qualitative data, enhancing the credibility and reliability of the findings.

### 2.5 Informed consent, confidentiality, and ethics approval

The PI and Co-PI of the study supported the submission and presentation of the proposal, tools, and consents at the Centre for Injury Prevention and Research, Bangladesh (CIPRB) Ethical Review Committee (ERC) meeting. The feedback from the ERC board members was incorporated into the proposal and resubmitted to the ERC, which subsequently provided final approval of the study.

## 3 Results

This section provides the results of the feedback from HCPs and stakeholders in key areas such as impact, coverage, cost-effectiveness, challenges, and sustainability of Vayu bCPAP. Furthermore, the section includes recommendations on Vayu bCPAP based on the results of the study (Table 2). Overall, the results section provides valuable insights for healthcare practitioners and policymakers on the usebility, acceptability, and usability of implementing Vayu bCPAP in healthcare facilities in Bangladesh.

**Table 2:** Distribution of interview and FGD responses according to thematic areas of the study.

Areas	Responses
Introduction of Vayu bCPAP	➢ Easy to assemble and portable. ➢ The device doesn't require electricity. ➢ Vayu bCPAP works with environmental air pressure, so a central oxygen supply is not necessary. ➢ Can be implemented in a facility with minimal resources.
Training and mentoring	➢ The training was hands-on skills and very effective. ➢ Mentors visited the hospital and addressed any issues raised. ➢ The coordination of mentors was very helpful.
Mentoring	➢ WhatsApp groups play a vital role in addressing problems faced by the users in any of the five facilities. ➢ It helps to address any issues quickly, leading to improved outcomes and patient satisfaction.
Impact of Vayu bCPAP	➢ The outcome is very good at an early stage of respiratory distress. ➢ The device has potential to reduce the need of mechanical ventilation. ➢ No perceived difference in terms of treatment outcome.
Coverage of Vayu bCPAP	➢ Referral numbers decreased after using Vayu bCPAP. ➢ Able to provide service to more patients. ➢ It improves the respiratory distress of babies.
Cost -effectiveness of Vayu bCPAP	➢ Vayu bCPAP can be operated with oxygen cylinder. ➢ Cost-effective for both the patient party and the hospital. ➢ Very effective in the case of term babies. ➢ Referral decreased after using Vayu bCPAP.
Challenges of implementation	➢ Stock out of consumables. ➢ Routine maintenance and cleaning. ➢ Continuous monitoring. ➢ Securing the position of nasal prongs and keeping this for a long time. ➢ If the flow increased slightly, the water overflowed and soaked the bed. ➢ The machine creates noise when it is in high airflow. ➢ Unavailability of nasal prongs. ➢ Accumulation of fog.
Sustainability of Vayu bCPAP	➢ Nurses need more training and continuous monitoring. ➢ Government can provide this to all levels of facilities after piloting. ➢ Compared to the “prior commercial bCPAP device” this one is very cost-effective.
Recommendations	➢ This device will be helpful in low-resource centers including sub-district level health care facilities. ➢ The consumables of the device should be available. ➢ Further study is required for the improvement of the device addressing any trouble-shooting needed.

### 3.1 Introduction of Vayu bCPAP as an alternative bCPAP device

Vayu bCPAP was introduced in the SCANUs as an alternative bCPAP device where availability of a commercial bCPAP was not adequate. The device has been considered to supplement the commercial bCPAP devices to provide support to a higher number of patients with RDS.

*“Before introducing the Vayu bubble CPAP, HCPs used the “prior commercial bCPAP device” for a long time. Then they came to know about a device that is easy to handle and is low-cost. It was on trial in different countries during the COVID-19, when a lot of people needed respiratory support*.” –Key informant interviewee

*“The Vayu device is an alternative to other bCPAP devices.” Unlike other bCPAP devices,” it doesn't require electricity to function. It is portable, easy to access, and cheaper in price.”* –FGD respondent

### 3.2 Training on Vayu bCPAP

Respondents noted that the training model was effective, including the facilitation and cascading of training.

*“After the orientation program, formal training was given at BSMMU where consultants from the SCANU of intervention facilities took part. In turn, they trained all other service providers of the SCANU including medical officers and nurses regarding the implementation of the Vayu bCPAP at facilities. Later, the supporting staff was given training by the nurses.”* –IDI respondent

*“The training was very effective as the researchers and engineers who were directly involved in the development of this device were present in the training and they trained them in a hands-on skill manner.”* –IDI respondent

### 3.3 Mentoring on Vayu bCPAP

According to respondents, during the early stages of implementing a new device, the training is mostly theoretical until it is put to use in practical settings. Once the device is being used, certain issues emerge that require attention and resolution. The master trainers of BSMMU played a crucial role in providing support to the users of Vayu bCPAP. They acted as a backup for addressing any issues that arose during the regular use of the device. This ensured that the devices were being used effectively and efficiently, which ultimately improved patient care. Additionally, the WhatsApp group maintained by the Save the Children staff and delegates of the five institutions played a vital role in addressing problems faced by the users in any of the five facilities. This group facilitated communication between users and provided a platform for sharing experiences and best practices. It helped to address any issues quickly, leading to improved outcomes and patient satisfaction (Table 2).

*“In the initial phase of introducing any device, the training to some extent remains theoretical until it starts to be used in practical fields. After starting to use the device, some problems arise which need to be addressed later. The master trainers of BSMMU act as back-up for addressing any issues arising during the regular use of the device.” –*Key informant interviewee

*“The WhatsApp group maintained by the Save the Children group and delegates of the five institutions play a vital role in addressing these problems faced by the users in any of the five facilities.” –*Key informant interviewee

*“The experts of BSMMU provide mentorship support regarding the usage of the machine. Their mentorship helped to address any issues that arise during the regular use of the device.” –*IDI respondent

### 3.4 Impact of Vayu bCPAP

The impact of Vayu bCPAP on newborns with respiratory distress was found to be positive. Vayu bCPAP was effective in managing large numbers of patients with RDS and in reducing the need for mechanical ventilation, which is associated with complications such as lung injury and infections. The study team found no perceived difference in the treatment outcomes (e.g., shorter hospital stays) of patients on Vayu bCPAP compared to those who received standard oxygen therapy (Table 2).

*“Unlike other bCPAP devices, it doesn't require electricity to function. It is portable, easy to access, and cheaper in price. Moreover, there is no perceived difference in the outcome between Vayu bCPAP and “prior commercial bCPAP devices.” –*FGD respondent

*“The Vayu bCPAP is an incredibly straightforward device that boasts a lightweight design, making it much easier to transport than its traditional counterparts. The device can be set up in a matter of seconds. Furthermore, this machine does not require electricity, making it even more convenient to use.”* –IDI respondent

*“As it is less expensive and doesn*’*t need electricity, it is more acceptable. It can blend oxygen and maintain pressure, but there is no perceived difference in terms of service outcome.”* –Key informant interviewee

### 3.5 Coverage of Vayu bCPAP

Respondents maintained that Vayu bCPAP is efficient and easy to use. The device requires minimal training, and healthcare providers can set it up quickly. Additionally, Vayu bCPAP is portable, making it easy to transport between facilities during referral. The device has potential for greater coverage and access, especially in rural areas where healthcare resources may be limited (Table 2).

*“When we started using the device, the level of confusion reduced and gradually the acceptance of the device increased in our facility.” –*FGD respondent

*“As a result of the introduction of the Vayu bCPAP, the healthcare facility is now able to provide support to a larger number of babies who require it.” –*Key informant interviewee

*“The existing good coordination among the BSMMU, DGHS, and the Save the Children team will be helpful for the successful implementation of the Vayu bCPAP and further scale up.” –*Key informant interviewee

### 3.6 Cost-effectiveness of Vayu bCPAP

Vayu bCPAP is a cost-effective alternative to prior commercial bCPAP devices, as well as mechanical ventilation, because the device is reusable, so costs are further reduced in the long term (Table 2).

*“Vayu bCPAP was introduced due to its cost-effectiveness. In Bangladesh, there is always some financial constraint in the utilization of modern technologies. The available commercial bCPAP devices are very expensive. So, when they found that the cost [of] Vayu bCPAP was very cheap and was given to them at zero cost initially, they took the opportunity to increase the quantity of CPAP machines to provide support to a greater number of babies requiring respiratory support.” –*Key informant interviewee

*“As there are financial constraints in our country, low-cost devices are given priority here. Besides, the other commercial bCPAP devices were not present in sufficient numbers in different facilities due to their high price. As the Vayu bCPAP is of low cost and easy to handle, it can be very effective if supplied to district-level hospitals. Babies requiring respiratory support will benefit from this device.” –*IDI respondent

### 3.7 Challenges of Vayu bCPAP in the facilities

One of the main challenges of implementing Vayu bCPAP identified by respondents is the need for regular maintenance and cleaning. The device requires periodic replacement of components, and healthcare providers need to be trained on how to properly clean and maintain it. Other key challenges highlighted by respondents include stock out of consumables, lack of a built-in warming system, loud noise and vibratory effect from the pressure generator, difficulty in securing the nasal prong, lack of humidity temperature recording, and the need for continuous monitoring, which is difficult due to inadequate staffing in SCANUs (Table 2).

*“Some challenges we faced in the initial phase were regarding the acceptability of the device as it is human nature to take time to adopt new things, and for having confusion regarding the maintenance of the temperature of the babies. The service providers were not so confident in using the device. However, when they started using the device, the level of confusion reduced and gradually the acceptance of the device increased in their facility.” –*Key informant interviewee

*“Other bCPAP devices have a monitor to see the temperature of the humidified air that helps in the maintenance of temperature which is not present in the Vayu one.” –*IDI respondent

*“Placing it in the baby*’*s cot may pose a problem as it takes up a considerable amount of space on the bed, which can be problematic for babies.” –*FGD respondent

*“The service providers were very happy as the Vayu bCPAP machine is very easy to use. They faced challenges when there was a shortage of consumables. Also, in the initial phase during the sterilization of reusable parts*.” *–*FGD respondent

### 3.8 Sustainability of Vayu bCPAP in the facility

According to respondents, Vayu bCPAP was considered a sustainable solution for addressing respiratory distress in infants. The device is portable and can be easily transported between facilities. The low cost and long-term reusability of the device make it an economically sustainable solution. Additionally, Vayu bCPAP has the potential to reduce the environmental impact of respiratory care, as it reduces the need for resource-intensive mechanical ventilation (Table 2). Respondents mentioned that the following should be done to sustain use of bCPAP: ensure there are proper baseline and endline results/research, ensure the proper awareness of the usage of the tool among the caregivers, build knowledge of dos/don'ts and troubleshooting among users, and make sure all stakeholders work together in implementation.

*“The equipment procurement cost, maintenance cost, accessories cost, cost for training of the service providers, and cost for further research should be considered for Vayu bCPAP use to be sustained in the facilities.” –*Key informant interviewee

*“The stakeholders are monitoring the program to improve the quality of service. However, for now, the focus is on learning the feasibility of this device, and if it is feasible and there is a positive outcome in the pilot phase, they will plan for its sustainability.” –*Key informant interviewee

*“The cost of consumables and the device should be made available and there should be a budget for it. Secondly, training and dissemination costs, and finally, maintenance costs. There should be a core biomedical group to maintain the device.” –*IDI respondent

### 3.9 Recommendations of Vayu bCPAP

Respondents had several recommendations related to Vayu bCPAP. They recommended that healthcare facilities incorporate Vayu bCPAP into their respiratory care protocols, and that healthcare providers receive training and follow-up support on the proper use, maintenance, and cleaning of the device. Additionally, they felt efforts should be made to increase the availability of Vayu bCPAP in areas where it is not currently accessible (Table 2). Respondents also recommended that:

• consumables be made available at the national level,
• maintenance of Vayu bCPAP should be made easier,
• devices should be available to manufacture locally,
• devices should be improved by adding a warming capacity,
• standard operating procedures should be developed,
• training should be ongoing, and
• availability of devices throughout the country should be increased.

*“It is essential to include and implement the Vayu bCPAP as a government program to sustain it beyond the pilot phase or to scale it to other similar facilities in Bangladesh. BSMMU has played a vital role in introducing and improving the device. However, more research activities on it are essential to gather evidence-based information on its effectiveness.” –*Key informant interviewee

*“First, the centers for implementation should be increased. Secondly, the results of the pilot phase should be shared with everyone involved in the implementation process. Comparative studies should be conducted regarding using Vayu bCPAP machine in different age groups in different conditions to learn about the outcomes. Then, training and capacity built-up. Lastly, providing the machine according to demand.” –*Key informant interviewee

## 4 Discussion

The introduction of a new medical device into regular health services, and adoption of the device by service providers and health facilities, requires an in-depth examination of its implementation in the local context (13–15). In our study, we investigated the useability and acceptance of Vayu bCPAP by program managers, service providers, and institutions, as well as the implementation challenges of using the Vayu bCPAP device in the local context of Bangladesh.

The study found that the Vayu bCPAP device is useable and acceptable in Bangladesh SCANU settings for the management of respiratory distress across all types of service providers. The device doesn't require electricity. The outcomes of use for early stages of respiratory distress were excellent, and referral numbers decreased after implementation of Vayu. The technical simplicity and easy bedside portability and assembly of the device enhanced the early initiation of respiratory support in SCANU settings and increased the service acceptability of the new device. These study results are generally consistent with the findings of other studies conducted in other similar settings around the world (16–19).

Our study also found that Vayu bCPAP systems were easily utilized by service providers across all levels of study facilities, were well-suited for the local context, and seamlessly integrated into the SCANU settings of Bangladesh. This easy utilization and seamless integration allowed nurses to initiate required treatment immediately. If needed, nurses were able to access distant support from a pediatrician using a virtual (WhatsApp) platform. These positive changes in RDS management in SCANU settings due to the introduction of Vayu bCPAP aligned with observations in other resource-constrained settings where simple CPAP devices were introduced (20, 21).

Several studies have outlined notable challenges associated with introducing CPAP devices in low- and middle-income countries (18, 22–24). Our study reveals the major challenges of Vayu bCPAP application in Bangladesh include stock out of consumables, the loud noise from the pressure generator, difficulty in securing the nasal prong, lack of a humidity temperature recording, and the need for continuous monitoring, which is difficult due to inadequate staffing in SCANUs. Continuous monitoring and maintenance of the equipment is crucial to ensure that it is being used effectively and safely, and to prevent any potential problems (25). Currently, there are no affordable oxygen blenders routinely available and used in many low- and middle-income countries, resulting in overuse of high oxygen concentrations and causing a near epidemic of Retinopathy of Prematurity, which can result in blindness or reduced vision among former preterm babies and children. Thus, the Vayu bCPAP system, especially in combination with the low-flow oxygen blender, offers substantial benefits for preterm survival and improved long-term outcomes in low-resource settings.

The study found that hands-on training is necessary to fully understand how to use and maintain the Vayu equipment properly. It is important to have someone show the health care providers how to use the equipment, followed by practice using it until one feels confident and comfortable (26).

The study also revealed that it is important to understand the setup and mechanism of the equipment to ensure the safe and effective operation of the device (27). This knowledge can help service providers to troubleshoot and fix any problems that may arise. The device is very low maintenance since there are no motors and it does not use electricity, so most maintenance can be done by trained service providers. The building of proper skills for maintenance enables service providers to use the device effectively, reducing infants' exposure to oxygen toxicity during recovery and as they are weaned off respiratory support and high concentrations of oxygen. This feature holds great promise to reduce the incidence and severity of Retinopathy of Prematurity and blindness in the most severe cases.

Basic maintenance and cleaning are essential to ensure that the equipment is functioning optimally and to prevent any contamination or damage to the equipment. Proper maintenance and cleaning also help to extend the lifespan of the equipment (28–30). While basic training is important, ongoing training is also necessary to ensure that service providers are confident and comfortable with the maintenance and cleaning of the equipment (31, 32). To successfully assemble and operate the equipment, the service providers need both knowledge and practice (33, 34). Therefore, continuous training, routine maintenance and cleaning, the ability to manufacture locally for rapid procurement, and development of standard operating procedures for the device, are mandatory components to provide quality services. In addition, reliable availability of consumables and adequate staffing for continuous monitoring are also crucial for desired service delivery and sustainability.

This study plays a vital role in building a body of evidence regarding the utilization of Vayu bCPAP within the specific healthcare context of Bangladesh. It not only addresses the perceptions of service providers and the challenges related to ensuring high-quality service delivery, but also highlights key recommendations for formulating a comprehensive service delivery model. Such a model can serve as a blueprint for effectively implementing Vayu bCPAP in other countries that face similar healthcare challenges as Bangladesh.

Nonetheless, it's important to underscore that further scientific research is required to comprehensively evaluate the implementation and impact of Vayu bCPAP in terms of reducing newborn and infant mortality rates nationwide.

## 5 Strengths and limitations

This qualitative research represents a pioneering effort which aimed to assess the useability and acceptability of the Vayu bCPAP device among healthcare service providers across various types of health facilities within the specific context of Bangladesh. The study conducted in-depth interviews involving key stakeholders, including national-level decision-makers, facility managers, pediatricians, relevant service providers, and supporting staff, to comprehensively understand the critical factors associated with the introduction of this new medical device to enhance service delivery in SCANU settings. By interviewing service providers and managers at different levels, the study sought to triangulate insights, challenges, and recommendations, thereby gaining a more holistic perspective. However, it is worth noting that this approach had an unintended drawback in that it created a perception among service providers that Vayu bCPAP was primarily introduced for research purposes rather than immediate patient care. The data collection process continued until data saturation was reached and interviewees expressed satisfaction with their participation. The interviewers were rigorously trained, and the composition of the interviewing team was suitably diverse.

It is crucial to point out some limitations of this study. Firstly, the relatively short implementation period may have limited the depth of service providers' perceptions, as forming a comprehensive understanding of a new device and its effects in facilities typically demands more time. Nevertheless, the high utilization of the Vayu bCPAP device during this implementation phase contributed to an adequate grasp of participants' experiences and perceptions. Secondly, the potential for researchers' subjectivity and biases may influence data collection and analysis. To address this, the study incorporated reflexivity and implemented measures to minimize potential bias.

## 6 Conclusion

Vayu bCPAP is a promising solution for respiratory distress in infants, with positive impacts on patient outcomes, cost-effectiveness, and sustainability. The challenges associated with the device can be addressed through proper training and distribution strategies. The recommendations provided based on this evaluation can guide healthcare facilities in incorporating Vayu bCPAP into their respiratory care protocols, ultimately leading to better outcomes for infants with respiratory distress. Regular monitoring, hands-on and ongoing training, understanding of setup, basic maintenance and cleaning, increased confidence and preparation, and knowledge and practice on Vayu bCPAP are all very important. Overall, the positive results of using Vayu so far demonstrate its effectiveness and ease of use. However, further scientific investigation is required to ascertain its non-inferiority compared to existing bCPAP devices and to address potential implementation and scalability challenges. Development of standard operating procedures for protocolized implementation is recommended.

## Data Availability

The raw data supporting the conclusions of this article will be made available by the authors, without undue reservation.
